# Warm versus cool colors and their relation to color perception

**DOI:** 10.1167/jov.25.4.13

**Published:** 2025-04-23

**Authors:** Jake Manalansan, Lorne A. Whitehead, Michael A. Webster

**Affiliations:** 1 Department of Psychology and Graduate Program in Integrative Neuroscience, University of Nevada, Reno, Reno NV, USA; 2 Department of Physics and Astronomy, University of British Columbia, Vancouver, BC, Canada

**Keywords:** color perception, color categories, saturation, warm–cool, uniform color spaces

## Abstract

The distinction between warm and cool colors is widely considered a fundamental aspect of human color experience, but whether it reflects properties of color perception or color associations remains unclear. We examined how the warm–cool division is related to perceptual landmarks of color coding and color appearance. Observers made warm–cool ratings for 36 hue angles at three luminance levels and also estimated the angles for their unique (e.g., yellow or red) and binary (e.g., orange) hues. The warm–cool dimension was reliably identified by most observers, was consistent across lightness levels, and varied along an orangish-red to greenish-blue dimension that is intermediate to both the principal chromatic dimensions of early cone-opponent (cardinal) or perceptual-opponent (red–green and blue–yellow) axes. When the stimuli were projected into a uniform color space (CIELAB), a close correspondence was found between the warm–cool dimension and the perceived strength (saturation) of different hues, based on the LAB chroma. Specifically, the peak warm and cool values were hue angles with the weakest saturation, and the boundaries between the two categories corresponded to hue angles with the highest saturation. This pattern could arise if vision is selectively adapted to the spectra of warm and cool colors and provides a potential basis for the strong but unexplained asymmetries in color coding built into perceptually uniform color spaces.

## Introduction

The wavelengths of light vary continuously, but our experience of color is punctuated by prominent divisions in how we represent and categorize the spectral content of stimuli. These divisions arise at many levels, from how neural mechanisms encode color to how we think about and interpret color. Physiologically, chromatic information is initially carried by the relative activity of the three classes of cone receptors, which absorb light with photopigments maximally sensitive to short (S), medium (M), or long (L) wavelengths of the visible spectrum. Subsequently, the signals from the cones are combined at post-receptoral stages to form a set of opponent dimensions. In the retina and lateral geniculate nucleus, the primary dimensions for color coding are given by the comparison of the L and M cones (LvsM) or signals in the S cones opposed by L and M cones (SvsLM) (Dacey, 2000; Derrington, Krauskopf, & Lennie, 1984). These cone-opponent dimensions are known as the “cardinal directions” of early color coding (Krauskopf, Williams, & Heeley, 1982). In visual cortex, the signals from these mechanisms are further transformed into “higher order” color mechanisms that are tuned to different directions in color space (Krauskopf et al., 1982; Lennie, Krauskopf, & Sclar, 1990), with the tuning becoming narrower at higher stages (Kiper, Fenstemaker, & Gegenfurtner, 1997). Further transformations are likely at later levels of the visual pathway, yet how color might be represented within these later cortical stages remains poorly understood.

The principal dimensions of color experience have also been inferred by phenomenological measurements of how colors appear. In classic color-opponent theory, different hues can be represented by the perceptual dimensions of red versus green and blue versus yellow (Hurvich & Jameson, 1957). These dimensions differ from the cardinal axes (Krauskopf et al., 1982; Webster, Miyahara, Malkoc, & Raker, 2000; Wuerger, Atkinson, & Cropper, 2005), and the neural underpinnings of the perceptual-opponent axes have yet to be resolved (Godat et al., 2024; Webster, 2020). Moreover, it is unclear whether they have the same status or basis as the physiologically defined dimensions. For example, the notion that a pure red or yellow sensation represents the isolated response of a red–green or blue–yellow mechanism is difficult to reconcile with the apparent population code for color in the cortex, in which any hue should stimulate multiple channels (Webster & Mollon, 1991; Webster & Mollen, 1994). Further, the perceptual dimensions that appear special could reflect special properties of the environment, such as the blue–yellow variation of daylight or surfaces, rather than special states of activity in the brain (Mollon, 2006; Pokorny & Smith, 1987). These issues have raised increasing doubts about the validity of conventional color-opponent models (Bosten & Boehm, 2014; Conway, Malik-Moraleda, & Gibson, 2023; Webster, 2020). Nevertheless, red–green and blue–yellow are still widely considered to be fundamental dimensions of color appearance.

At still another level, colors can be classified along a wide range of dimensions that may reflect preferences, semantic correlates, or emotional impact (Elliot & Maier, 2014; Palmer & Schloss, 2010). These can include experiencing colors as light versus heavy, active versus passive, clean versus dirty, or like versus dislike (Gao et al., 2007; Ou, Luo, Woodcock, & Wright, 2004). Dimensions of this kind may correspond more closely to color cognition and to the associations we make to different colors and thus may be based on very different principles from the underlying neural and perceptual codes. For example, much of the variance in color preferences can be accounted for by the valences for the object categories people associate with different colors (e.g., blue with clear sky and water, brown with dirt or rotting fruit) (Palmer & Schloss, 2010). Yet, on the other hand, sex differences in color preferences have been found to reflect different weightings of the cardinal (LvsM) mechanisms, illustrating the interplay of multiple levels in shaping color judgments (Hurlbert & Ling, 2007).

In this study, we focused on one of the most prominent yet least understood dimensions of color experience: warm versus cool. This distinction is widely invoked in studies of the impact or emotion of color (Ou et al., 2004); yet, it is also frequently considered a fundamental dimension of color appearance (Hardin, 2000; Katra, Wooten, & Knoblauch, 2023; Koenderink & van Doorn, 2021) and has been tied to aspects of color perception ranging from neural color coding (Mollon, 1989; Rosenthal, Singh, Hermann, Pantazis, & Conway, 2021) to color communication (Gibson et al., 2017; Lindsey & Brown, 2006) to salient signatures of the color environment (Rosenthal et al., 2018). The status of warm–cool judgments—and in particular the level(s) of representation to which they refer—is thus uncertain (Knoblauch, Werner, & Webster, 2023; Koenderink & van Doorn, 2021; Koenderink, van Doorn, & Braun, 2024b; Zaslavsky, Kemp, Tishby, & Regier, 2019). Here, we asked how warm–cool ratings align with perceptual landmarks of human color vision. Although the warm–cool axis is distinct from the dimensions of cone-opponent (cardinal axes) or perceptual-opponent (unique hues) processes, the division shows a strong and surprising alignment with the relative saturation of different colors, a pattern that may reflect interactions across multiple levels of color experience.

## Methods

### Participants

Participants included authors JM and MAW and 24 students from the University of Nevada, Reno (UNR). Participation was with informed consent, and all procedures followed protocols approved by UNR's institutional review board.

### Apparatus and hardware

Experiments were presented on a MultiSync FP2141SB CRT monitor (NEC Corporation, Tokyo, Japan) through a ViSaGe board (Cambridge Research Systems, Kent, UK), which provides high color resolution. The monitor was calibrated using a PR-655 spectroradiometer (Photo Research, Chatsworth, CA) with linearized gun outputs. Participants used a handheld keypad to record their responses.

### Stimuli

The chromaticities of the stimuli were defined within a variant of the MacLeod–Boynton (MacLeod & Boynton, 1979) and Derrington–Krauskopf–Lennie (Derrington et al., 1984) color spaces. These spaces represent chromatic signals in terms of the two cone-opponent axes (LvsM and SvsLM) at constant luminance. The two axes were scaled so that unit distances along each axis corresponded roughly to multiples of threshold, based on previous studies (Webster & Mollon, 1994). The coordinates of the stimuli were given by

$$\begin{array}{c} \mathrm{LvsM}=1955*\;(l_{\mathrm{mb}}-0.6568) \end{array}$$

$$\begin{array}{c} \mathrm{SvsLM}=5532*\;(s_{\mathrm{mb}}-0.01825) \end{array}$$

where l_mb_ and s_mb_ are the coordinates in the MacLeod–Boynton diagram, and 0.6568 and 0.01825 are the coordinates of the neutral gray, which had a chromaticity equivalent to illuminant C. For the experiments, the chromatic contrast was fixed at a value of 80 while the hue angle varied, with an angle of 0° corresponding to the +L pole of the LvsM axis and 90° to the +S pole of the SvsLM axis. The background had a fixed luminance of 20 cd/m^2^, and the test stimulus had luminance values of 10 cd/m^2^, 20 cd/m^2^, or 40 cd/m^2^. The stimuli were viewed binocularly from a distance of 2 meters in an otherwise dark room.

### Procedure: Warm–cool judgments

**Figure 1.**
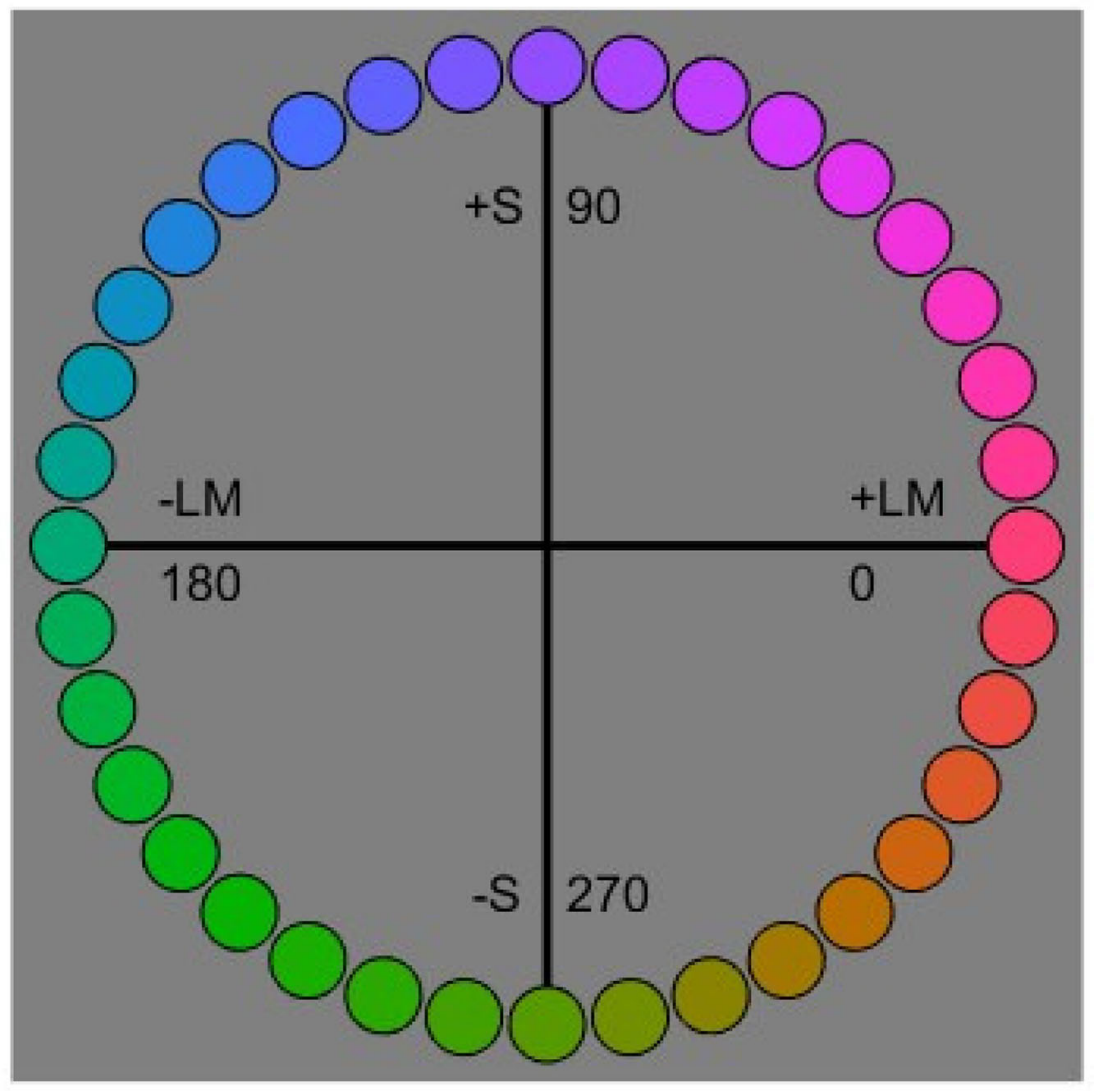
Color space for the experiment, defined by variations in LvsM cone or SvsLM cone signals at constant luminance. Stimuli had a fixed chromatic contrast and varied in angle relative to the neutral gray.

**Figure 2.**
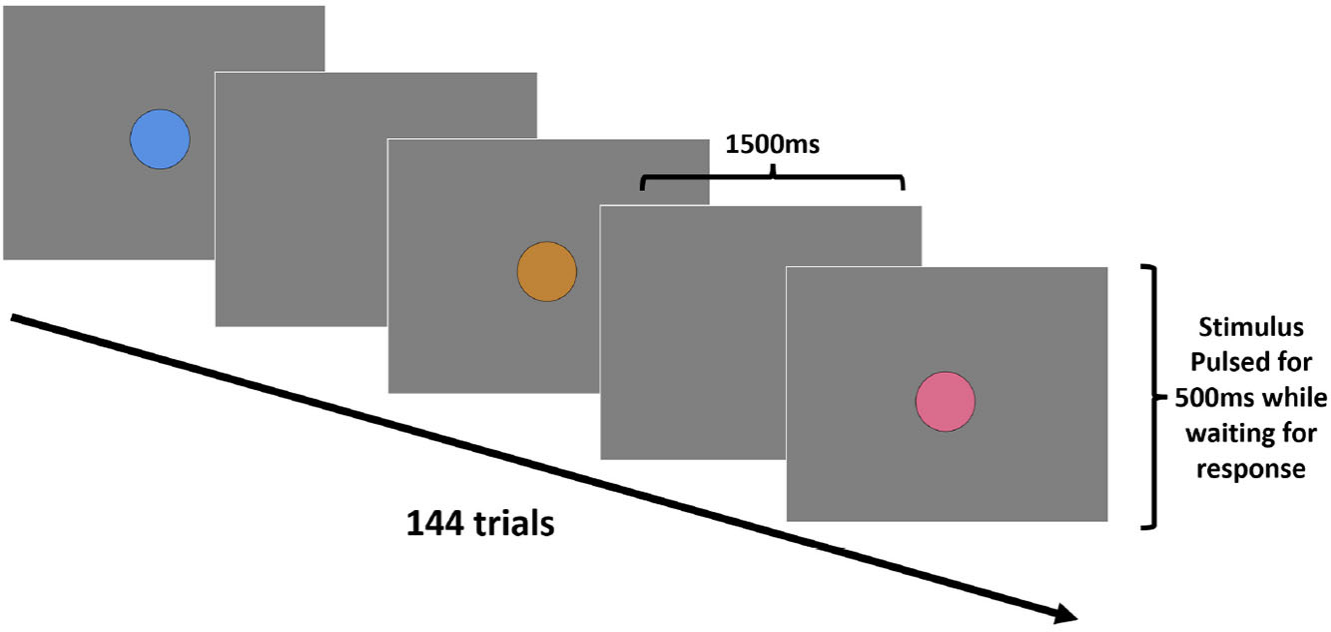
Experimental procedure for the warm–cool judgments. Each color was presented in random order and pulsed for 500 ms on, 1500 ms off until the participant rated the warm–cool value using a seven-point scale.

For the main experiment, colors consisted of 36 hue angles spanning the LvsM and SvsLM plane in steps of 10° (Figure 1). These were presented sequentially in random order in a uniform 2° circular field centered on the 8° × 6° gray background corresponding to the screen size of the display (Figure 2). The stimulus was pulsed on for 500 ms with an interstimulus interval (ISI) of 1500 ms and continued until a response was made, after which a new random hue angle was shown. The observer responded by using a seven-point Likert scale to rate how warm or cool the color appeared (with categories of very warm, warm, somewhat warm, neutral, somewhat cool, cool, and very cool). During the run, each hue angle was shown four times in random order at the same luminance level, and each session included runs at the three different luminance levels (10, 20, and 40 cd/m^2^), which were counterbalanced for order across observers. Reported values for individual observers are based on the average of the four repeated settings per condition.

### Procedure: Unique and binary hues

**Figure 3.**
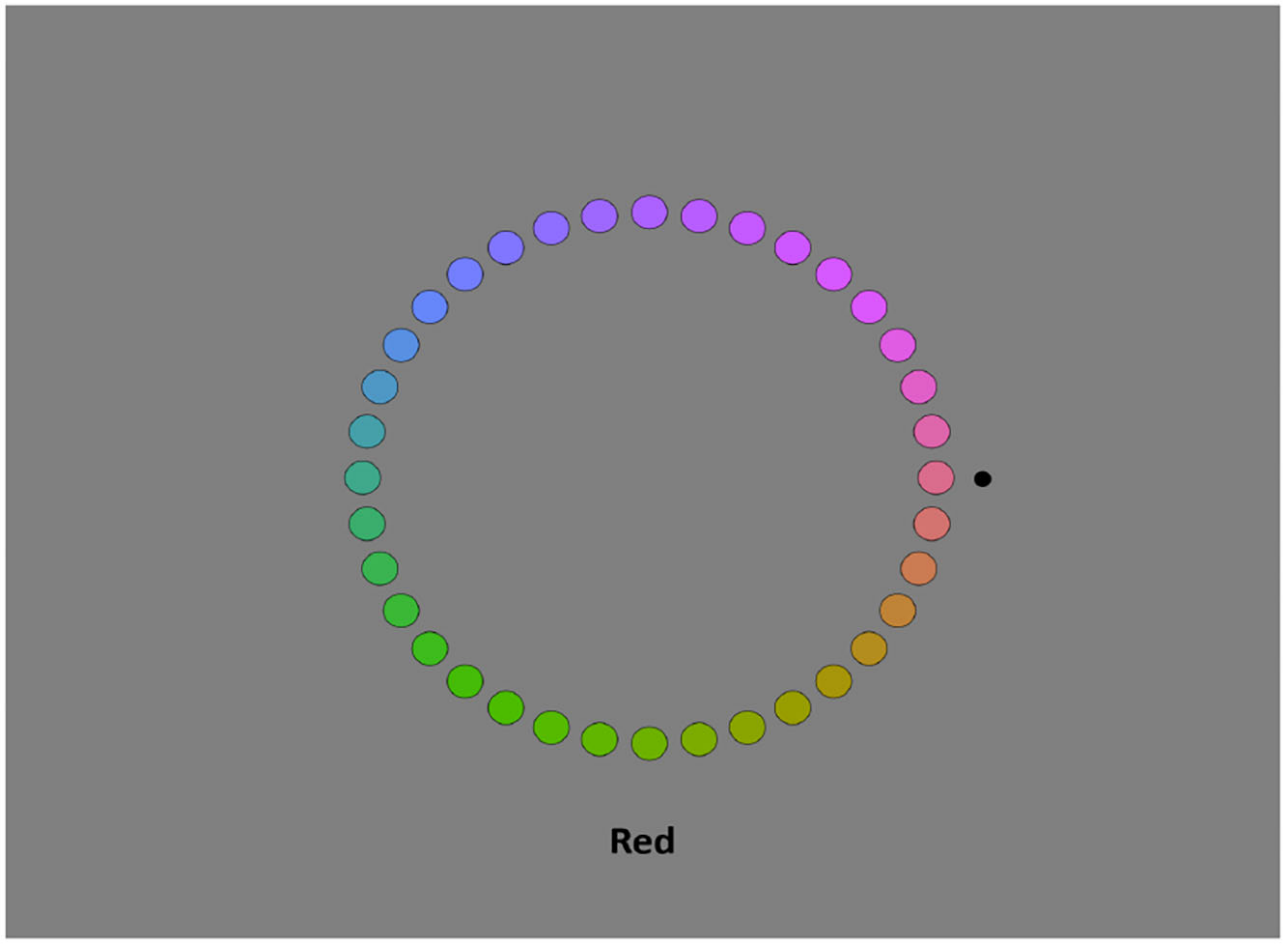
Illustration of the experiment for choosing unique and binary hues. The black dot was moved clockwise or counterclockwise to select the angle corresponding to the cued text (red in this example). Note that participants were instructed to estimate the best angle, which could be intermediate to the displayed colors.

In an ancillary experiment, for each observer we also estimated the hue angles corresponding to the unique hues (pure red, green, blue, or yellow) and binary hues (purple, cyan, yellow–green, and orange, which correspond to mixtures of the unique hues). In this case, the entire set of colors was displayed on the gray background, with each circle subtending 0.75° and with a luminance of 20 cd/m^2^ (Figure 3). The participant used the keypad to move a pointer clockwise (6 on the number pad) or counterclockwise (4 on the number pad) around the hue circle to indicate the angle of each hue, and the color term was displayed in black at the bottom of the screen. Participants were instructed to estimate the precise hue angle and not simply choose the closest example from the hues displayed. They were also instructed to locate the hues based on the null point (e.g., neither red nor green for unique yellow or blue) or the equal balance for binary hues (e.g., equal amounts of red and yellow for orange). Prior studies have shown that unique hue judgments also correspond closely with ratings of the “best example” or focal color of each hue (Miyahara, 2003). On each run, the observer made settings for each color term in random order and for four repetitions of each, with the cursor position randomized before each trial. Two repeated runs were made during the session, and the individual data reported are based on the mean of the eight settings.

## Results

### Warm–cool ratings and their relation to hue and lightness

**Figure 4.**
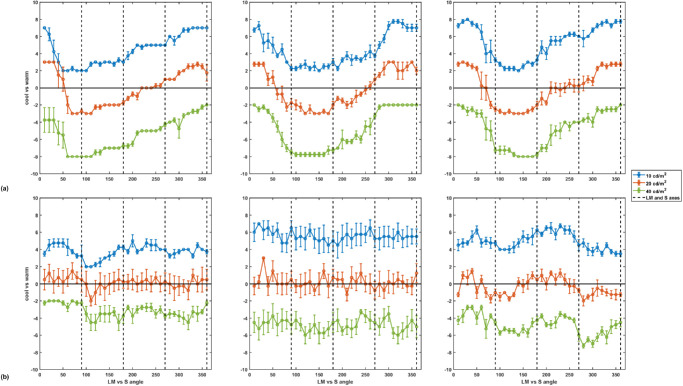
Representative individual ratings for warm versus cool colors. Each panel shows the mean ratings for warm versus cool (±1 *SEM*) on a seven-point scale (–3 cool to +3 warm) as a function of the stimulus angle in the LvsM and SvsLM chromatic plane. The three curves show the settings at each luminance level and are arbitrarily shifted vertically by +3 (10 cd/m^2^ targets), 0 (20 cd/m^2^), or –3 (40 cd/m^2^) for clarity. (**a**) Top panels illustrate typical results from three of 19 observers whose warm–cool ratings systematically varied with chromatic angle. (**b**) Bottom panels show results from three of six observers who were excluded from subsequent analyses because their settings did not exhibit a consistent warm–cool chromatic axis. Dashed vertical black lines show the LvsM and SvsLM axes of the color space.

Settings for representative individual observers and are shown in Figure 4. Based on these settings, 19 of the 25 observers systematically classified the different hues consistent with a single and roughly continuous warm versus cool dimension and were therefore included for further analyses (Figure 4, top panels). The remaining six were excluded because their settings showed either very low or non-systematic variation with hue, unimodal responses, or multiple apparent peaks for warm or cool (Figure 4, bottom panels). Thus, these observers either were insensitive to the warm–cool distinction or used it to classify the hues in qualitatively different ways. Similar differences between observers have also been reported in other studies of the warm–cool dimension (Devinck & Knoblauch, 2025; Katra et al., 2023; Koenderink et al., 2024b).

**Figure 5.**
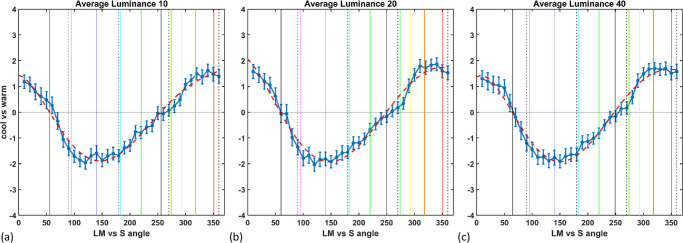
(**a**–**c**) Average warm–cool ratings (blue solid line and points) and polynomial fit (red dashed line) for targets at the three luminance levels indicated. Note that the fitted line shows the function used to estimate the cool minimum and warm–cool boundaries. A separate fit (not shown) was used to estimate the warm peak (after phase shifting the data by 180° so that the peak fell closer to the center of the measurement range). Points show the mean across observers ± 1 *SE*. Colored vertical lines represent the mean values for the eight hue loci, dashed vertical lines represent the LM and S axes, and solid vertical lines represent the warm–cool boundaries.

Figures 5a to 5c plot the average functions for the 19 observers who exhibited a consistent pattern. The three panels plot the mean settings at the three lightness levels. As previous studies have found, on average the warm–cool division varies roughly from orangish-red (warm) to greenish-blue (cool) (Devinck & Knoblauch, 2025; Hammond, Gardner, Wooten, & Renzi-Hammond, 2024; Katra et al., 2023; Koenderink & van Doorn, 2021; Koenderink et al., 2024b). This dimension thus differs from both the cone-opponent axes (LvsM and SvsLM) and the canonical red–green and blue–yellow dimensions of color appearance. In particular, the functions are consistent with the general finding that the perceptual primaries of red and yellow are both warm colors, but the primaries blue and green are both associated with cool colors (Hardin, 2000; Katra et al., 2023; Knoblauch et al., 2023), although, as shown below, in our settings the warm and cool peaks were closer to red and blue than to orange and blue–green.

To estimate the actual peaks and boundaries of the functions, we fit a fifth-order polynomial (using the MATLAB function polyfit; MathWorks, Natick, MA) to both the average settings and to each observer's individual settings, and we used these to determine the angle of the zero crossings (boundaries) or the maxima (warm peaks) or minima (cool peaks) of the functions. Because the warm peaks tended to be close to the limits of the 0 to 360 range of the functions, to estimate these peaks a separate fit was made after phase-shifting the function 180° (so that the values ranged from –180 to 180). As shown by the dashed lines in Figures 5a to 5c, which plot the fits for the minima and zero-crossings, the polynomials provide a reasonable fit for estimating the values.

**Figure 6.**
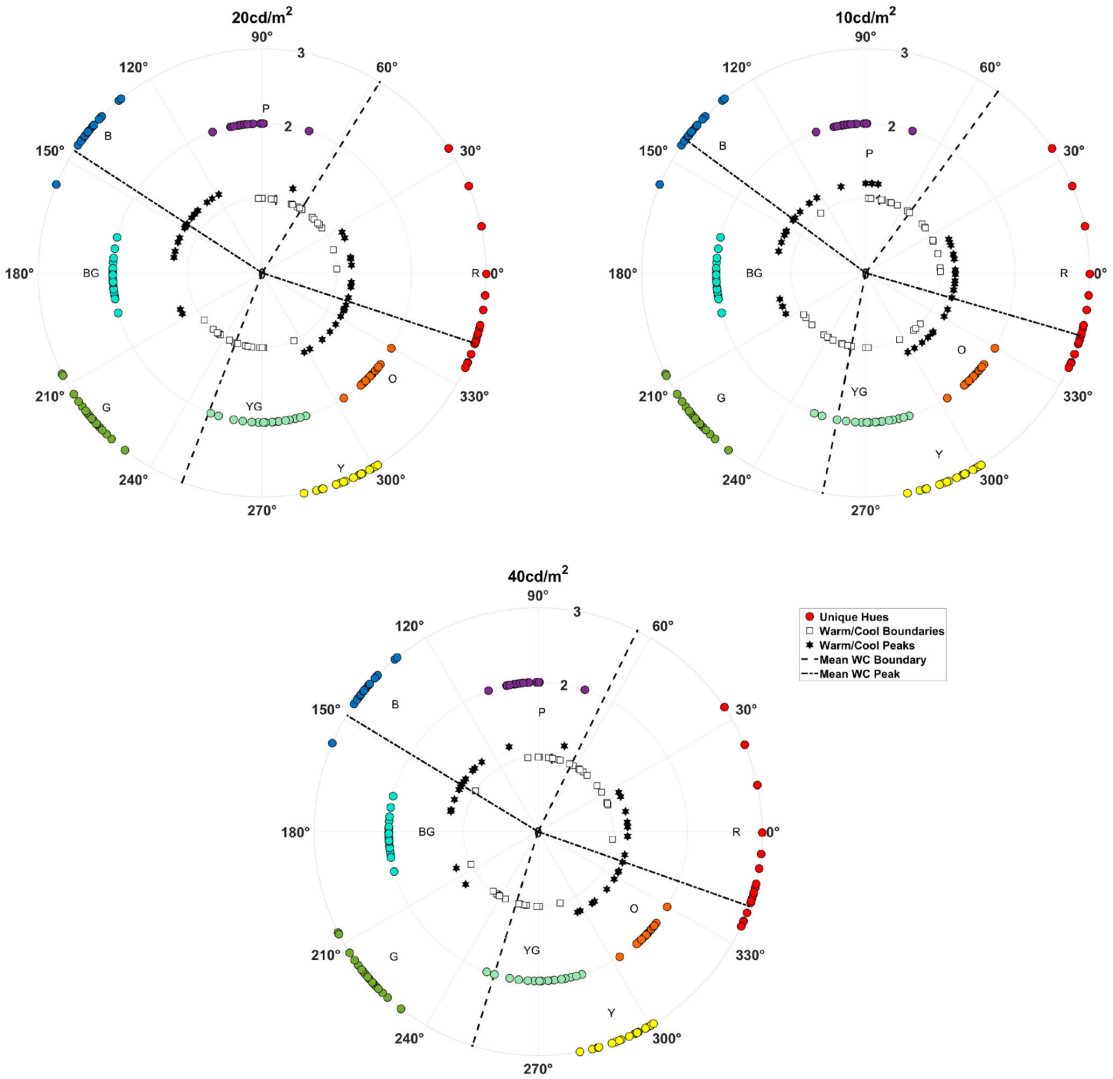
Individual warm–cool boundaries (unfilled symbols, innermost radii) or peaks (second radii), compared to the focal choices for the unique and binary hues, as indicated by the category labels. Dashed lines indicate the average angles for the warm–cool peaks (small dashes) and boundaries (large dashes). The three panels show the settings for the three lightness levels. Note that unique and binary hue settings were only collected at 20 cd/m^2^ and are repeated across the panels.

Figure 6 plots the warm–cool peaks and boundaries for each of the observers (innermost points), along with the color angles selected for the unique and binary hues (see also Table 1). The three panels again show the settings for the three lightness levels tested (with the hue loci again tested only at 20 cd/m^2^ and therefore duplicated across lightness levels). These results point to the following properties of warm–cool judgments. First, the warm–cool functions were similar across the different lightness levels. This is consistent with previous findings of Katra et al. (2023), though two recent studies have found some effects of lightness on the judgments (Devinck & Knoblauch, 2025; Hammond et al., 2024).

**Table 1.** Mean angle and standard deviation of the warm–cool peaks and boundaries and of the eight hue loci.

	µ ± σ		
	10 cd/m^2^	20 cd/m^2^	40 cd/m^2^
Warm	343.7 ± 27.4	341.7 ± 25.3	340.5 ± 27.6
Cool	143.5 ± 36.1	146.9 ± 30.5	148.7 ± 30.2
Boundary 1	53.9 ± 32.1	58.4 ± 24.3	63.7 ± 31.2
Boundary 2	258.9 ± 31.3	249.1 ± 18.7	252.9 ± 30.4
Yellow	—	292.7 ± 5.7	—
Blue	—	139.9 ± 5.9	—
Green	—	220.2 ± 6.5	—
Red	—	350.2 ± 16.1	—
Blue–green	—	181.8 ± 6.9	—
Orange	—	316.1 ± 5.5	—
Purple	—	95.5 ± 7.6	—
Yellow–green	—	272.1 ± 10.3	—

Second, there are large individual differences in both the warm–cool functions and the observers’ selections for the color terms. This inter-observer variability is a consistent finding in studies of color appearance, although the basis for it remains poorly understood (Emery, Volbrecht, Peterzell, & Webster, 2023; Emery & Webster, 2019; Kuehni, 2004; Lindsey & Brown, 2006; Lindsey & Brown, 2009; Webster et al., 2000). The variations in the warm–cool settings were highly consistent across lightness levels. Correlations between settings across the three luminances averaged 0.93 for the warm peaks and 0.89 for the cool peaks, and they were also strong for the warm–cool boundary in the “purple” direction (∼60°) or warm–cool boundary 1 (averaging 0.84), although they were generally weak for the second warm–cool boundary in the “yellowish-green” direction (∼260°) (see Table 2). These high correlations suggest that that the differences among observers were reliable.

**Table 2.** Correlations between the same warm–cool peaks or boundaries across the three luminance levels. Corrections for multiple comparisons here and elsewhere are based on the Benjamini–Hochberg procedure for controlling the false discovery rate.

	10 vs. 20 cd/m^2^	10 vs. 40 cd/m^2^	20 vs. 40 cd/m^2^
Warm peak	0.91^*^	0.95^*^	0.94^*^
Cool peak	0.89^*^	0.82^*^	0.96^*^
Warm–cool boundary 1	0.79^*^	0.96^*^	0.76^*^
Warm–cool boundary 2	0.84^*^	0.22	0.30

* *p* ≤ 0.05 corrected for the 30 multiple comparisons reported in Tables 2 and 3.

In contrast, at each lightness level the variations in the peaks and boundaries were more weakly correlated (Table 3) and also showed generally weak correlations with the observers’ hue foci, which were themselves not strongly correlated even for adjacent color categories (Table 4). The weak relationship between the loci for different hues is consistent with previous studies based on larger sample sizes (Emery et al., 2023; Webster et al., 2000), although the power of the present study to assess these relationships is more limited because of the smaller sample. In the case of the warm–cool dimension, there was a trend for the warm or cool peaks to covary with the purplish warm–cool boundary, but the correlations between warm and cool, or between the two boundaries, were not themselves significant. The average warm and cool peaks also did not significantly differ from the mean settings for red and blue, respectively, *t*(18) ≤ 1.32, *p* ≥ 0.20, but were nevertheless uncorrelated with them. Taken together, these results suggest that the warm–cool dimension is distinct from other color categories, and moreover that the dimension itself does not reflect a single underlying process (because warm and cool tended to vary independently). This is again similar to the findings for red–green and blue–yellow dimensions of color appearance for which the opposing primaries also vary independently and thus are not yoked as part of a common opponent mechanism (Emery et al., 2023; Webster et al., 2000).

**Table 3.** Correlations between warm–cool peaks or boundaries at each luminance level.

	10 cd/m^2^	20 cd/m^2^	40 cd/m^2^
Warm vs. cool	0.50	0.41	0.23
Warm vs. boundary 1	0.59^*^	0.83^*^	0.51^*^
Warm vs. boundary 2	0.20	0.23	−0.24
Cool vs. boundary 1	0.64^*^	0.49	0.67^*^
Cool vs. boundary 2	0.48	0.73^*^	0.36
Boundary 1 vs. 2	−0.07	0.13	−0.07

^*^ *p* ≤ 0.05 corrected for the 30 multiple comparisons reported in Tables 2 and 3.

**Table 4.** Correlations between the warm–cool loci and hue loci at 20 cd/m^2^.

	Luminance 20 cd/m^2^							
	Red	Purple	Blue	Blue–green	Green	Yellow–green	Yellow	Orange
Red	1	—	—	—	—	—	—	—
Purple	−0.02	1	—	—	—	—	—	—
Blue	0.06	−0.06	1	—	—	—	—	—
Blue–green	−0.18	−0.15	0.04	1	—	—	—	—
Green	−0.20	−0.17	−0.23	0.10	1	—	—	—
Yellow–green	−0.47	−0.13	0.10	−0.01	0.53	1	—	—
Yellow	0.05	0.16	0.06	−0.14	0.04	0.14	1	—
Orange	−0.34	−0.40	−0.08	0.32	−0.01	0.14	−0.38	1
Warm–cool 1	0.46	−0.13	−0.20	−0.09	−0.20	−0.46	0.31	−0.54
Warm–cool 2	0.23	0.19	0.11	−0.61	−0.27	−0.05	0.26	−0.12
Warm	0.30	−0.11	−0.07	−0.24	−0.42	−0.48	0.37	−0.31
Cool	0.31	−0.04	−0.12	−0.40	−0.15	−0.12	0.20	−0.22

^*^ *p* < 0.05 corrected for the 60 multiple comparisons reported in Table 4.

Finally, the results also indicate that the warm–cool dimension is not aligned with the LvsM and SvsLM cardinal axes. In particular, at each lightness level the peaks of the function were significantly different from the cardinal axes, again with warm rotated toward orange relative to the +LM pole and cool rotated toward blue for the –LM pole, all *t*(18) ≥ 2.59, *p* ≤ 0.018. Similarly, the boundaries at each lightness were also significantly shifted toward red relative to the +S pole and toward green relative to the –S pole, *t*(18)>2.44, *p* ≤ 0.025, with the exception of the yellowish-green boundary at the 40-cd/m^2^ luminance, which was not significant, *t*(18) = 1.54, *p* = 0.14. Moreover, variations in the angles of the cardinal axes occur because of variations in spectral sensitivity, these are generally too small to account for variations in color appearance and also predict highly correlated changes across the opposite poles (Simoncelli & Webster, 2024; Webster et al., 2000), which again was not observed.

### Warm–cool ratings and their relation to saturation

To further explore the nature of warm–cool judgments, we next examined how they depended on the saturation of the different hues tested. The stimuli we sampled all had equal chromatic contrast as defined by distance from the neutral gray in the cone-opponent plane (as defined by our scaling metric). However, these distances do not necessarily have equal “perceived” chromatic contrast, or saturation. To explore this factor, we re-examined the settings within a different color space designed to capture the perceptual differences between colors rather than the cone-opponent signals defining the stimuli. For this, we projected the set of stimuli into the 1976 CIELAB uniform color space, which is a standard space for evaluating perceptual color differences (Luo, 2023). Such uniform color spaces apply nonlinear transformations of the color coordinates so that distances within the space (known as delta E) denote the magnitude of perceptual differences. All such uniform color spaces are known to provide only an approximation to perception but capture general and common properties of perceptual scaling. For example, the CIELAB representation was designed to be similar to the perceptual scaling of color predicted by the Munsell Color System, which is also a widely used and empirically based system for assessing color appearance and perceptual differences (Robertson, 1977).

**Figure 7.**
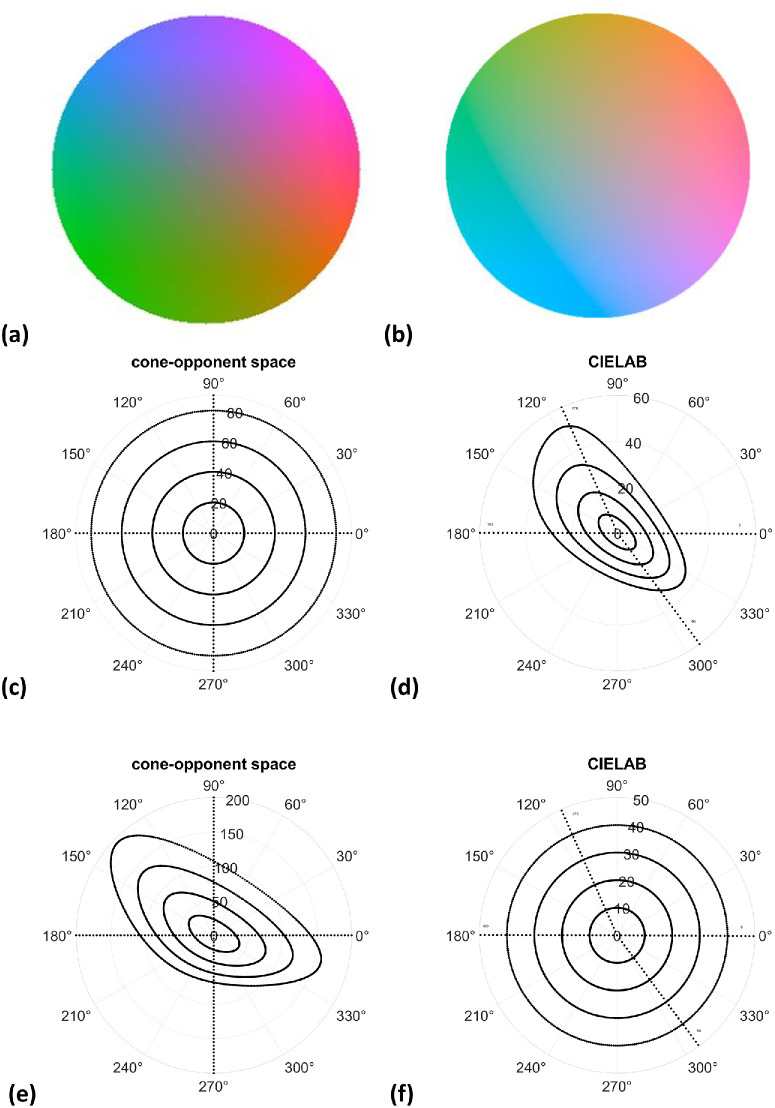
(**a**–**f**) Comparisons of equivalent stimulus contours in spaces defined by cone-opponent signals (**a**, **c**, and **e**) or scaled for perceptual strength (in CIELAB, **b**, **d**, and **f**). Signals with constant cone-opponent contrast (**c**) project to stimuli with different predicted saturation (**d**), and vice versa, so that constant CIELAB chroma (**f**) project to distorted cone-opponent contrasts (**e**). Dashed lines in all figures represent the cardinal directions in the cone-opponent plane. Note that our version of the cone-opponent space follows the MacLeod–Boynton diagram in plotting the S (90°–270°) axis as increasing S signals, which is inverted in the CIELAB space.

Figure 7 illustrates the relationship between stimuli in the cone-opponent and CIELAB spaces. The images at the top depict the location of different hues within each space. Contours with uniform cone-opponent contrast (Figure 7c, as in our stimulus set) project to distorted contours in CIELAB (Figure 7d), with points closer to the CIELAB origin corresponding to weaker (lower chroma or less saturated) stimuli. Conversely, contours with constant chroma in the CIELAB (Figure 7f) require different magnitudes of cone-opponent contrast for different hues and thus again project to distorted contours in the cone-opponent diagram (so that the weakest hues require larger cone contrasts for the same perceptual magnitude) (Figure 7e).

**Figure 8.**
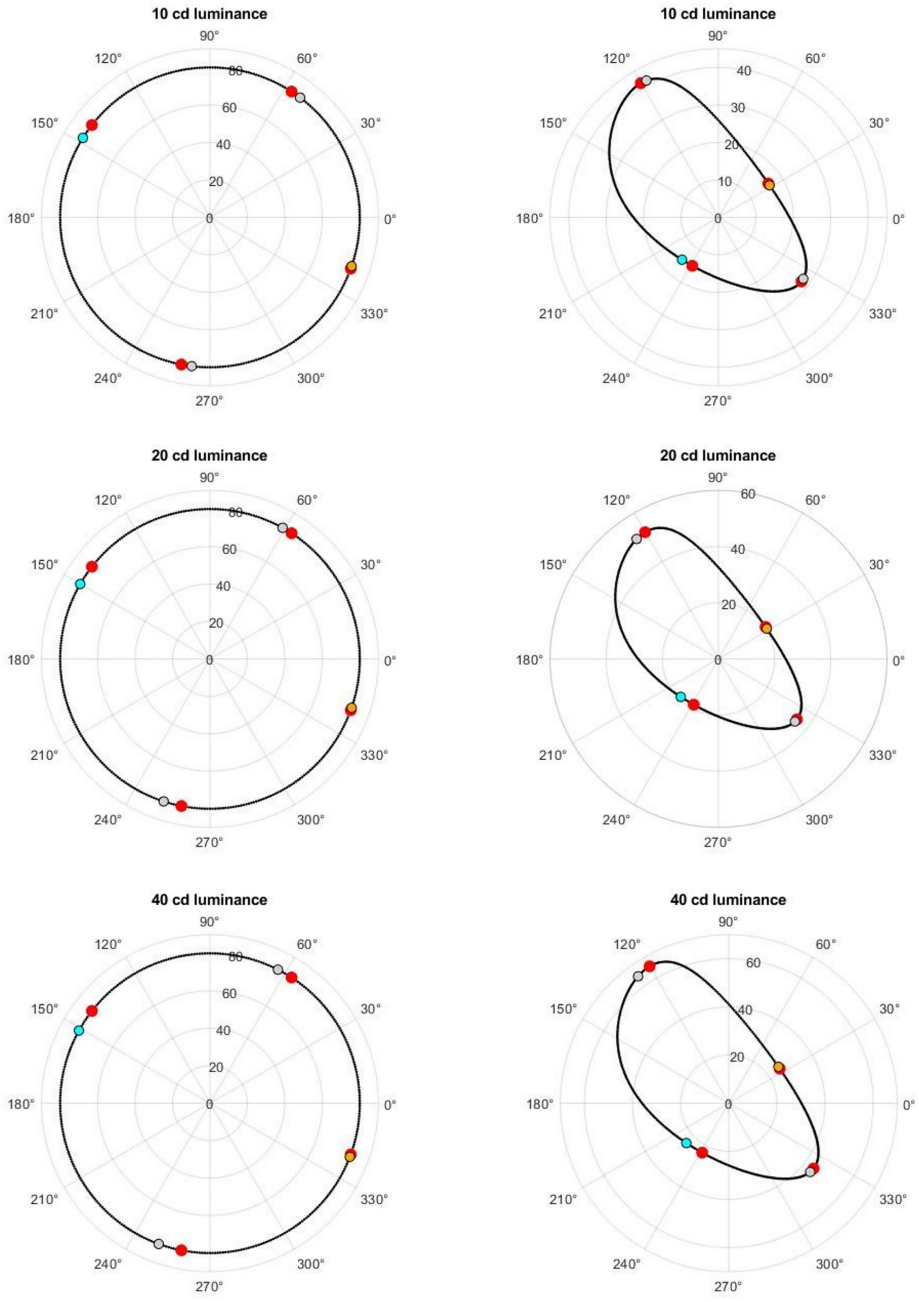
Stimulus sets in the cone-opponent space (left panels) or CIELAB uniform color space (right panels) for the three lightness levels tested. Each panel compares the warm peak (orange), cool peak (cyan), or warm–cool boundaries (gray) to the maxima and minima of the saturations for the constant cone-opponent contrasts in the uniform color space (red).

Figure 8 shows the cone-opponent contour we sampled for the three lightness levels and their projection into CIELAB. For each we estimated the maxima and minima of the projected contours (shown by the red symbols in the figure) and compared these to the warm and cool peaks and boundaries based on the fits to the average warm–cool functions (shown by the orange, cyan, and gray symbols, respectively, in the figures). This analysis revealed a striking correspondence between the orientation of the warm–cool dimension and the orientation of constant saturation within the cone-opponent space.

**Figure 9.**
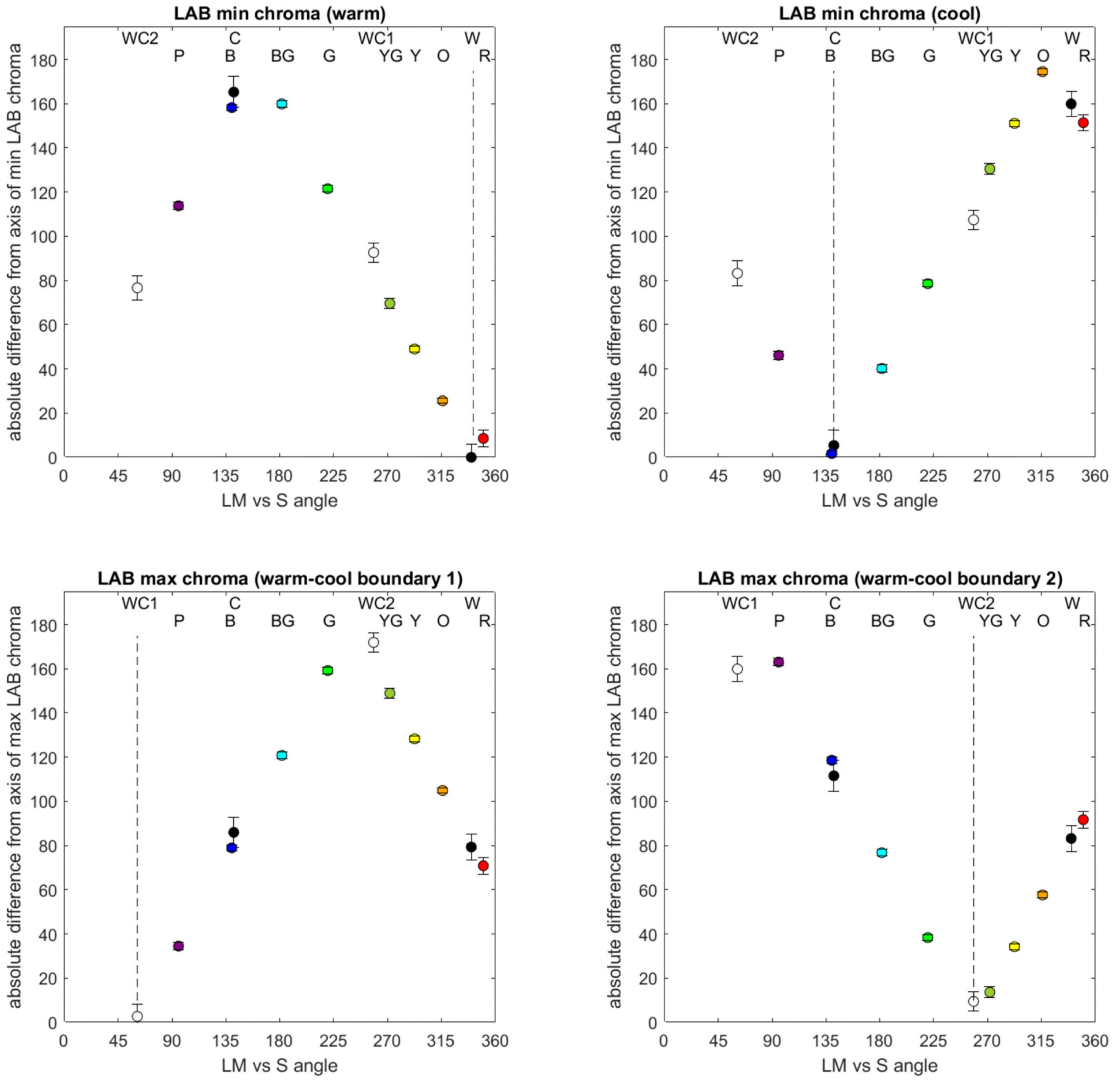
Correspondence between the warm–cool or hue loci and the saturation maxima and minima predicted by the CIELAB chroma of the stimuli. Symbols plot the absolute angular difference between each measured hue angle (given by the labels at the top of each panel) and the angles of each of the four extrema of the LAB contours (shown by the dashed vertical lines). Error bars: ±1 *SEM*.

This is further shown in Figure 9, which plots the absolute difference between the angles (in cone-opponent space) of the warm–cool or hue settings versus the angles of the four maxima and minima from the projected CIELAB contours (all for the luminance of 20 cd/m^2^). In each case, the warm–cool peaks or boundaries fell very close to the maximum or minimum values in the contours and did not significantly differ from them (except for WC2, which was not significant after correcting for multiple comparisons). In contrast, all of the hue foci were significantly different from the contour extrema, *t*(18) ≥ 2.73, *p* ≥ 0.014, with the exception of unique blue, *t*(18) = 1.26, *p* = 0.22, which was similar in angle to the cool peak.

A further important feature of this association is that the hues that are strongest in the warm–cool dimension are in fact the weakest in saturation; that is, the warm–cool peaks correspond to the minima in saturation contours, whereas the boundaries correspond to the maxima. Notably, these associations would not be apparent if stimuli were analyzed within either color space alone, but are instead revealed by the relationship between the physiological cone-opponent contrasts and their perceptual strength as predicted by standard uniform color spaces.

**Figure 10.**
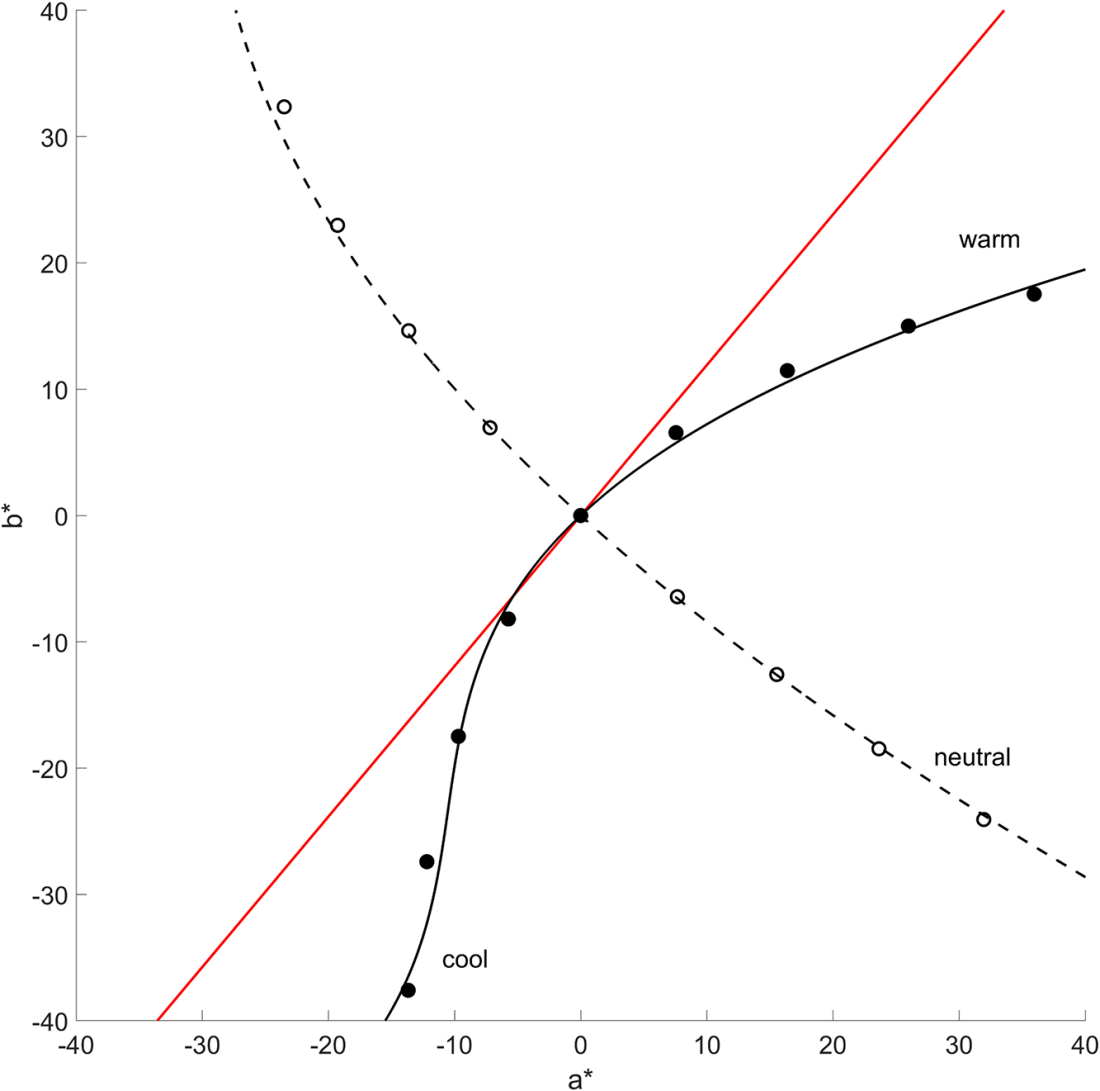
Predicted warm–cool axis (solid black line) and boundary (dashed line) from the minima (filled symbols) and maxima (unfilled symbols) of iso cone-opponent contrast contours within the CIELAB space. The solid red line plots the warm–cool dimension estimated by Ou et al. (2004).

These saturation loci and our findings that these loci closely track the warm–cool dimension can provide a useful heuristic for predicting the “heat” of colors, something that is of wide interest in color applications (Ou et al., 2004). To construct this, we estimated the cone-opponent maxima and minima over a range of constant-chroma values within the LAB space and then fit these as a function of the A and B color coordinates of the stimulus. The contours are depicted in Figure 10, along with the warm–cool dimension modeled by Ou et al. (2004), which represents a simple linear axis in LAB along an angle of 50°. The dimension based on the saturation contours predicts a roughly similar direction in the color plane but is strongly curved. By this account, the equations for predicting the warm–cool axes and strength for a given A–B coordinate are

$$\begin{array}{ccc} & & \;\text{Warm-cool}\;\;\text{axis}\;\;A\;=\;1.0674B\;+\;0.0396B^{2}\;+\;0.000565B^{3}\\ & & \;\text{Warm-cool}\;\;\text{boundary}:\;\;A=-1.0996B+0.0104B^{2}\\ & & \;\text{Warm-cool}\;\;\text{strength}:\;\;S_{\mathrm{wc}}=0.6792A+0.7233B\\ & & \;+\;0.0013A^{2}+0.0042\mathrm{AB}+0.0044B^{2} \end{array}$$

## Discussion

As noted, the notion of warm versus cool colors has been central to discussions of color experience at many levels, ranging from principles of color appearance to art to the philosophy of color (Hardin, 2000; Katra et al., 2023; Knoblauch et al., 2023; Koenderink & van Doorn, 2021; Koenderink, van Doorn, & Braun, 2024a; Koenderink et al., 2024b; Levine, 1991; Ou et al., 2004; Palmer, 1999; Unwin, 2012). Many previous studies have measured warm–cool scales for stimuli, and our results are largely consistent with these in showing that the dimension encompasses red and yellow as warm colors and blue and green as cool, a partition that is intermediate to both the cardinal LvsM and SvsLM axes of early cone-opponent coding and to the red–green and blue–yellow axes of color appearance (Hardin, 2000; Katra et al., 2023; Knoblauch et al., 2023). The idea that it represents a distinct dimension is further suggested by our findings that individual variations in the warm–cool settings were independent of variations in focal color choices. This suggests that there may be only a weak link between the warm–cool foci and specific hues. Moreover, as with different hue categories, we also found that the warm and cool foci were themselves uncorrelated. Again, this was despite the fact that warm–cool loci remained very consistent across the different lightness levels, indicating that the interobserver variations in the settings were highly reliable relative to the within-observer settings. This suggests that it may be inaccurate to characterize warm and cool as two poles of a single underlying opponent dimension, just as red and green (or blue and yellow) do not appear strongly tied to a unitary opponent process (Emery et al., 2023; Webster et al., 2000). Instead, warm and cool colors may reflect two largely complementary yet independent categories. But what do these categories represent?

In the World Color Survey, a monumental study of color naming patterns across 110 languages (Kay, Berlin, Maffi, Merrifield, & Cook, 2009), some languages have been shown to classify all colors with only two basic color terms that group colors roughly in terms of a warm–cool divide, and Kay and McDaniel (1978) therefore suggested that this division could be the earliest stage in the evolution of linguistic color categories (Kay & Maffi, 1999). This superordinate dimension has also been found in statistical analyses of the naming patterns within the World Color Survey. When these patterns are limited to two clusters, the responses across languages divide in terms of a warm-color dimension, and the lowest concordances in naming are for stimuli near the warm–cool boundary, suggesting that stimuli on either side of the boundary are treated as distinct (Lindsey & Brown, 2006). Consistent with this evidence for warm versus cool categories, Holmes and Regier (2017) also found categorical perception along the warm–cool dimension, such that within-category stimuli (e.g., two warm colors) were less distinct than stimuli that spanned the category boundary.

Such studies reveal the prominence of potential warm and cool categories but leave open the question of their basis. However, color naming patterns have also been analyzed in terms of the information they communicate (Jameson, 2005). Across languages with varying numbers of basic color terms, the terms associated with warm colors are communicated more efficiently (Gibson et al., 2017; Lindsey, Brown, Brainard, & Apicella, 2015). Analyses of the chromatic information in images have also found that warm colors are more strongly associated with objects but cool colors are more likely to be tied to the image background (Gibson et al., 2017; Rosenthal et al., 2018). This raises the possibility that the warm–cool dimension may be superordinate because it reflects a basic distinction between the relevance of color-associated information in scenes, although the warm–cool asymmetry may also reflect asymmetries in the structure of perceptual color space (Zaslavsky et al., 2019).

The link of warm and cool with scene color statistics could provide a potential account for our primary finding: that the warm–cool categories closely track the relative saturation of hues that are matched for equivalent cone-opponent contrasts (based on their relative chroma in CIELAB). Recall again that this relation was such that the hues that are most prototypical in terms of the warm or cool attribute reflect colors that are predicted to be weakest in saturation. Although this seems paradoxical for color categories, it is in fact consistent with the idea that color vision (like most if not all sensory processes) adapts to the gamut of stimuli to which the observer is exposed (von der Twer & MacLeod, 2001; Webster, 1996; Webster, 2014; Webster, 2015; Webster & Mollon, 1997). For example, natural daylight—and the color gamuts of many natural scenes—tend to vary most along a bluish-yellowish dimension, and, in turn, sensitivity to this dimension tends to be weaker (Bosten, Beer, & MacLeod, 2015; Goddard, Mannion, McDonald, Solomon, & Clifford, 2010; Skelton, Franklin, & Bosten, 2023; Skelton et al., 2024; Webster & Mollon, 1997). This has been explained by adaptation processes that match sensitivity to the stimulus range, so that the range of available neural responses remains optimized (Laughlin, 1981; von der Twer & MacLeod, 2001). Uniform color spaces might embody these long-term adaptations, so that the distortions in the relative perceptual salience of different hues (as illustrated in Figures 7 and 8) are inversely related to the strength of the corresponding color signals in the environment.

However, a conundrum has been that the biases in natural scene statistics do not in fact closely align with the biases predicted by uniform color metrics (McDermott & Webster, 2012). Specifically, the principal axes of natural color gamuts tend to be along bluish-yellowish axes for panoramic or arid scenes (Webster & Mollon, 1997), whereas, for scenes dominated by foliage, the distributions instead are biased toward the SvsLM cone-opponent dimension (i.e., toward greenish-yellow axes) (Regan et al., 2001; Ruderman, Cronin, & Chiao, 1998; Sumner & Mollon, 2000). In contrast, the saturation biases implicit in uniform color spaces are instead rotated toward orange and red (i.e., closer to the LvsM dimension). Thus, by this account, the salience of colors predicted by uniform color metrics has remained perplexing (McDermott & Webster, 2012).

The present results suggest a resolution to this discrepancy. The finding that the predicted salience corresponds closely to the warm–cool dimension—and that warm–cool colors correspond to a selective loss in perceived contrast along this dimension—suggests the possibility that visual coding is somehow adapted to the color variations along this dimension, rather than the more greenish-yellow biases found in the gamut of typical natural color distributions. One way this could occur is because global color statistics, on which most color statistics are based, do not take into account how observers visually sample scenes and the colors that are most important to attend to, such as the diagnostic colors of objects (Gibson et al., 2017). It may also be that the color distributions that humans in modern societies are adapted to are in fact unnatural, because they include fabricated colors in which reddish and orangish hues are likely far more prevalent than in typical natural scenes of forests and savannahs. Finally, most measures of the color statistics of scenes are for daylight and under-represent the reddish-orange lighting at dawn or dusk, which may have disproportionate influence on behavior (Dominy & Melin, 2020). Future studies might explore these questions by directly measuring the visual diet of modern environments as actually sampled by observers (Greene et al., 2024), and recently we explored the visual salience of the warm–cool axis by using a color search task (Manalansan, Simoncelli, & Webster, 2025). This showed that the warm–cool targets are less conspicuous compared to the orthogonal (∼warm–cool boundary) axis, but similar biases were also found for the blue–yellow axis. In any case, the current results suggest that the warm–cool dimension is not merely a conceptual construct but is tied to a prominent yet largely hidden and previously unexplained asymmetry in the perceptual processing of color, and one that might arise from and shed further light on how color appearance is shaped by the color environment.
